# The Effects of Carotid Pathologies on Short-Term Functional Outcomes After First-Ever Small Vessel Occlusion Stroke

**DOI:** 10.3390/brainsci15070773

**Published:** 2025-07-20

**Authors:** Minwook Bae, Yong-Suk Jeong, Sopheak Phoung, Phoeuk Borei, Dahyeon Koo, Dougho Park

**Affiliations:** 1 Department of Cardiology, Pohang Stroke and Spine Hospital, Pohang 37659, Republic of Korea; bae8078@gmail.com (M.B.); lukejungmd@gmail.com (Y.-S.J.); 2 Department of Cardiology, Khmer Soviet Friendship Hospital, Phnom Penh 12306, Cambodia; sopheak.peak@gmail.com (S.P.); phoeukboreicardio@gmail.com (P.B.); 3 Medical Research Institute, Pohang Stroke and Spine Hospital, Pohang 37659, Republic of Korea; 4 Medical Science and Engineering, Graduate School of Convergence Science and Technology, Pohang University of Science and Technology, Pohang 37673, Republic of Korea

**Keywords:** carotid intima–media thickness, carotid stenosis, lacunar stroke, ischemic stroke, prognosis

## Abstract

**Background:** While carotid pathologies are well-established risk factors for stroke, their specific effects on outcomes following stroke that cannot be classified as atherosclerotic remain unclear. In this study, we aimed to determine whether carotid pathologies are associated with functional dependence (FD) 3 months after small vessel occlusion (SVO) stroke. **Methods:** This retrospective study included patients with a first-ever SVO stroke admitted to a single cerebrovascular-specialty hospital between October 2021 and March 2024. Standardized ultrasound criteria were used to grade the carotid plaques. The modified Rankin scale (mRS) was used to assess functional outcomes at 3 months. Logistic regression analysis was performed to identify FD predictors (mRS of ≥2). **Results:** Of the 372 included patients, 276 achieved functional independence and 96 experienced FD at 3 months. Univariable analysis revealed an association between carotid plaque grade III and FD (odds ratio [OR], 2.46; 95% confidence interval [CI], 1.05–6.51; *p* = 0.049). However, this association was not significant in the multivariable model. Overall, age (adjusted OR, 1.07; 95% CI 1.03–1.10, *p* < 0.001), NIHSS at initial presentation (adjusted OR, 1.84; 95% CI, 1.55–2.18; *p* < 0.001), and diabetes (adjusted OR, 2.84; 95% CI, 1.37–5.92; *p* = 0.005) were independently associated with FD 3 months after SVO stroke. **Conclusions:** Carotid plaque severity was not independently associated with functional outcomes 3 months after SVO stroke. Age, NIHSS at initial presentation, and diabetes were identified as independent FD predictors. Future in-depth studies are warranted to confirm the complex interplay of factors influencing functional outcomes in patients with SVO stroke and carotid pathologies simultaneously.

## 1. Introduction

Small vessel occlusion (SVO) stroke, characterized by the occlusion of small penetrating arteries deep within the brain, accounts for approximately 20% of all ischemic strokes [1]. This subtype commonly results in lacunar infarcts, causing various lesion location-dependent clinical syndromes [2]. Despite being associated with favorable short-term outcomes, SVO stroke can still result in significant long-term disability, impacting the quality of life and independence of affected patients [3]. In addition, although SVO stroke presents lower initial severity compared with large artery atherosclerosis stroke, patients with SVO stroke can still suffer from progressive white matter damage, recurrent strokes, and vascular dementia [4].

Carotid pathologies represent significant risk factors for stroke. They have been extensively evaluated in the context of large artery atherosclerosis and their association with stroke risk and recurrence, particularly when associated with high-grade stenosis or vulnerable plaques [5,6]. However, the role of carotid pathologies in SVO stroke remains less well understood. Although the results of some studies indicate that carotid atherosclerosis may contribute to SVO occurrence through hemodynamic mechanisms or by acting as a source of emboli, other authors have identified no significant association [7,8]. In addition, although traditionally considered a distinct entity, certain authors posit that carotid intima–media thickness (CIMT) and plaque burden may be surrogate markers of generalized small vessel disease rather than sole indicators of embolic risk [9,10]. This ambiguity highlights the need for further in-depth studies into how carotid pathologies specifically influence SVO stroke outcomes.

Despite the increasing number of stroke-related studies, no consensus has been reached to date concerning the influence of carotid pathologies on functional outcomes in this specific stroke subtype. There is therefore an urgent need to better understand the relationship between carotid plaque severity and functional recovery after SVO stroke to enable risk stratification and the development of targeted interventions to optimize patient outcomes.

In this context, we aimed to investigate whether carotid pathologies can serve as predictors of functional outcome at 3 months after a first-ever SVO stroke. To achieve this aim, we analyzed a well-characterized cohort from a single cerebrovascular specialty hospital in South Korea.

## 2. Materials and Methods

### 2.1. Patient Inclusion

In this single cerebrovascular-specialty hospital study, we retrospectively included patients admitted with a diagnosis of SVO ischemic stroke between October 2021 and March 2024. All patients were also registered in the Korean Stroke Registry [11]. This study was reviewed and approved by the institutional review board (approval number: PSSH0475-202409-HR-017-01). All patients provided informed consent for the use of de-identified data before their registration in the Korean Stroke Registry (project management number: PSSH0475-201901-HR-001). We conducted this study in adherence with the Declaration of Helsinki.

**Figure 1:**
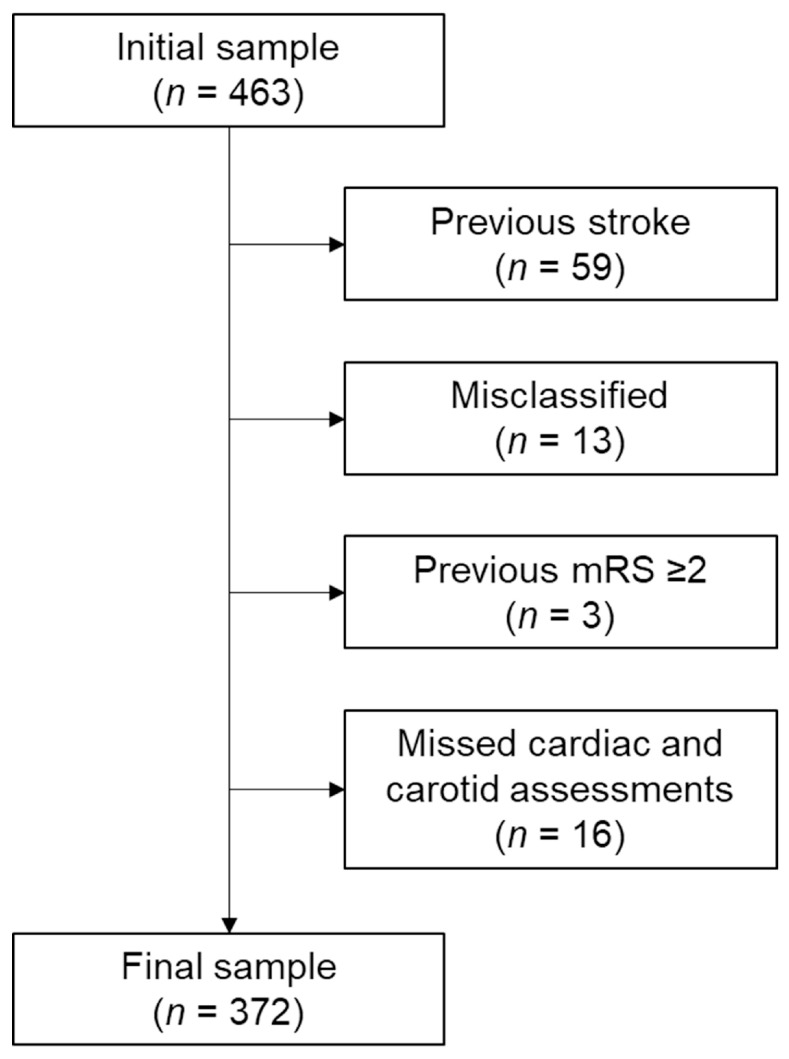
Flowchart of patient inclusion. Abbreviation: mRS, modified Rankin scale.

The initial sample included 463 patients. We determined the SVO type based on the Trial of ORG 10172 in Acute Stroke Treatment classification [12]. The diagnosis of SVO was established based on both clinical presentation—typical lacunar syndromes without cortical signs—and radiological confirmation of subcortical infarcts less than 20 mm in diameter on brain magnetic resonance imaging, which served as the gold standard for stroke subtype classification. To ensure diagnostic accuracy, we included only patients with neurological deficits persisting for more than 24 h. Experienced neurologists and neurosurgeons determined the final classification. The exclusion criteria included (1) stroke history identified by medical record, history taking, or imaging findings (n = 59), (2) vague or misclassified stroke type (n = 13), (3) previous disability (modified Rankin scale [mRS] ≥ 2; n = 3), and (4) missed cardiac and carotid assessments (n = 16). Ultimately, 372 patients were analyzed after applying the exclusion criteria (Figure 1).

### 2.2. Carotid Assessments

**Figure 2:**
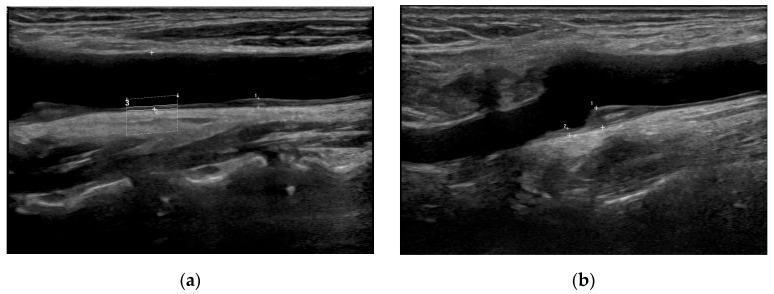
Representative carotid ultrasound images. (**a**) Grade I carotid plaque in the left common carotid artery. A small protuberant plaque is visualized with intima–media thickness (IMT) < 1.5 mm. (**b**) Grade III carotid plaque in the left internal carotid artery bulb. A large, protruding plaque with IMT ≥ 2.5 mm is noted, indicating high-grade carotid pathology.

We used a LOGIQ Fortis™ device with an L3–12 linear probe (GE Healthcare Technologies, Inc., Chicago, IL, USA) for the ultrasonographic carotid assessments. We measured the CIMT 10 mm proximal and distal to the carotid bifurcation (Figure 2) [13]. We classified the carotid plaques into three grades following the updated recommendations for carotid arterial plaque assessment by ultrasound from the American Society of Echocardiography: grade I, protuberant plaques with CIMT of <1.5 mm; grade II, either protuberant or diffuse plaques with CIMT ≥ 1.5 mm and <2.5 mm; and grade III, CIMT of ≥2.5 mm [14]. Cardiologists with more than 10 years of experience completed the carotid assessments. In patients with bilateral stroke lesions, we used the worst values. For patients with unilateral stroke lesions, CIMT and plaque measurements obtained from the carotid artery ipsilateral to the infarction side were used. In cases with bilateral stroke lesions, the more severe CIMT and plaque grade values were used for analysis.

### 2.3. Functional Outcome and Covariates

We used the mRS at 3 months as the primary functional outcome measure. We conducted the assessment at the outpatient clinic or via telephone follow-up 3 months from the date of hospitalization, with a permissible window of ±1 month. We analyzed the current functional level of the patient, the degree of support required for daily activities, the spatial extent of daily living, whether the pre-morbid standard of living was being maintained, and return to work. Based on the 3-month mRS, we categorized the patients into two groups: functional independence (FI) (3-month mRS 0 and 1) and functional dependence (FD) (3-month mRS ≥ 2) groups.

In this study, we used the Korean Stroke Registry guidelines to define covariates and provided the details in the online Supplementary Content (Document S1).

### 2.4. Statistical Analysis

We present the continuous variables as the means ± standard deviations and the categorical variables as frequencies (proportions). We used an independent *t*-test to compare continuous variables between the two groups and a chi-squared (trend) test for categorical variables. To predict FD 3 months after the index stroke, we performed univariable logistic regression followed by multivariable analysis by adding age and sex as covariates. We performed all analyses using R software version 4.4.1. (R Foundation for Statistical Computing, Vienna, Austria).

## 3. Results

### 3.1. Baseline Characteristics

Table 1 presents the baseline characteristics of the patients. Of the total patients, 276 and 96 were categorized into the FI and FD groups, respectively. The mean age of the FD group was 72.6 ± 11.7 years, with this value being significantly higher than the mean age of 66.6 ± 11.6 years in the FI group (*p* < 0.001). The proportion of males was higher in the FD (53.1%) than in the FI (33.7%; *p* = 0.001) group. The initial National Institutes of Health Stroke Scale (NIHSS) score was significantly higher in the FD than in the FI group (4.5 ± 3.2 vs. 1.7 ± 1.7; *p* < 0.001), and the NIHSS score at discharge also showed a significant difference (3.3 ± 2.6 vs. 0.5 ± 0.9; *p* <0.001). Regarding lesion location, the FD group contained a higher proportion of corona radiata lesions; in comparison, the FI group contained a relatively higher proportion of thalamic and posterior circulation lesions (*p* = 0.003). The proportion of grade III carotid plaques in the FD group was significantly higher (*p* = 0.016). No significant differences in the initial laboratory findings were observed between the two groups (Table S1).

The results presented in Table S2 demonstrate that there were no significant differences in mean CIMT or plaque grade distribution between males and females (*p* = 0.584 and *p* = 0.586, respectively). Similarly, the results presented in Table S3 illustrate carotid pathology characteristics stratified by stroke lesion location, revealing no significant differences in CIMT or plaque grade distribution across the different lesion sites (*p* = 0.624 and *p* = 0.219, respectively).

### 3.2. Logistic Regression Models

**Figure 3:**
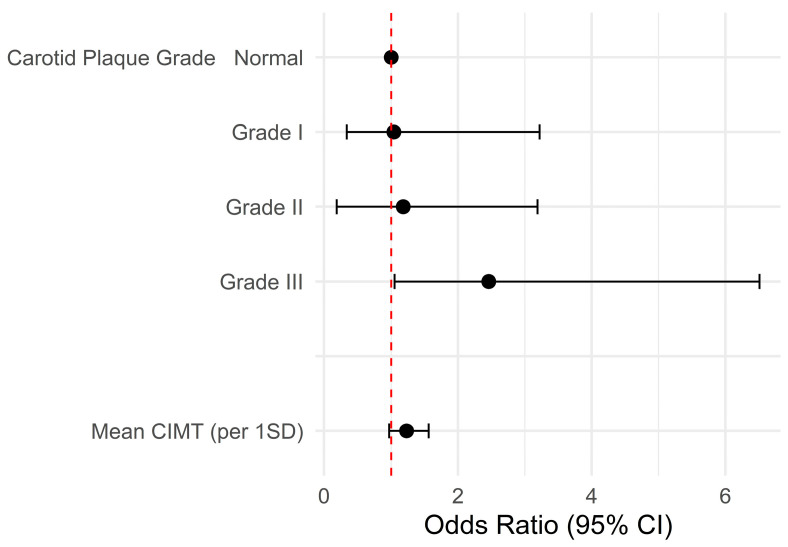
Forest plot of the univariable logistic regression model. The univariable model demonstrates the association between grade III carotid plaques and FD. Abbreviations: CI, confidence interval; CIMT, mean carotid intimal thickness; SD, standard deviation.

The logistic regression model revealed that the mean CIMT was not associated with FD after SVO. The univariable model demonstrated the association between grade III carotid plaques and FD (odds ratio [OR], 2.46; 95% confidence interval [CI], 1.05–6.51; *p* = 0.049) (Table 2 and Figure 3). However, the carotid plaque grades did not exhibit any statistical significance in the multivariable model. Age (adjusted OR, 1.03; 95% CI, 1.00–1.05; *p* = 0.047) and male gender (adjusted OR, 2.31; 95% CI, 1.36–3.97; *p* = 0.002) were associated with FD after a SVO (Table 3).

To further identify significant predictors of FD 3 months after SVO stroke, we performed a multivariable logistic regression analysis using a backward elimination method. The final model revealed age (adjusted OR, 1.07; 95% CI 1.03–1.10, *p* < 0.001), NIHSS at initial presentation (adjusted OR, 1.84; 95% CI, 1.55–2.18; *p* < 0.001), and diabetes (adjusted OR, 2.84; 95% CI, 1.37–5.92; *p* = 0.005) as independent predictors of FD. In this model, male gender (adjusted OR, 0.48; 95% CI, 0.24–0.93; *p =* 0.030) and thalamic lesions (adjusted OR, 0.14; 95% CI, 0.03–0.79; *p =* 0.026) were associated with a lower risk of FD (Table 4).

## 4. Discussion

In this study, we evaluated the factors affecting functional outcome 3 months after SVO stroke, primarily focusing on the effect of carotid pathologies. While the results of the univariate analysis indicate a potential association between carotid plaque grade III and FD, this finding did not persist in the multivariate model. In contrast, age, NIHSS at initial presentation, and diabetes emerged as independent FD predictors following SVO. Unlike previous related studies, our study focused exclusively on patients with a first-ever SVO stroke, enabling us to isolate the effects of carotid pathologies in this specific stroke subtype and deepen our understanding of SVO stroke mechanisms. Furthermore, our study has key advantages in that it presents the results of a well-defined patient cohort derived from a single cerebrovascular-specialty hospital and the Korean Stroke Registry, ensuring detailed clinical and imaging data.

The authors of previous studies have evaluated the relationship between carotid atherosclerosis and stroke outcomes [15], primarily focusing on large artery atherosclerosis and recurrent stroke risk [16,17,18]. However, the specific effects of carotid pathologies on functional outcomes in SVO stroke remain elusive. Our findings indicate that while severe carotid plaques might contribute to SVO stroke, their influence on functional outcomes may be less pronounced than that of other factors, such as age and gender. This finding may reflect the complex interplay of various factors, including stroke severity, lesion location, and individual patient characteristics, in determining functional recovery after SVO. Age has a well-established impact on post-stroke functional recovery, often outweighing other vascular risk factors. Older adult patients exhibit reduced neuroplasticity and impaired adaptive cortical reorganization, which may limit their functional recovery regardless of carotid pathologies [19]. In addition, males exhibited inconsistent results in our logistic regression models, which only partially supports the results of previous studies in which men were generally found to have poor functional outcomes [20]. In addition, stroke severity (measured based on NIHSS) is among the strongest predictors of long-term disability, often eclipsing the role of atherosclerosis-related factors in recovery prognosis [21]. Lastly, the authors of several studies have highlighted that lesion location significantly influences recovery potential, with corona radiata and internal capsule infarcts associated with more significant motor impairment and functional dependency [22,23]. Based on our results, a higher proportion of FD patients had basal ganglia and corona radiata lesions, which might have contributed more significantly to the poorer outcomes compared to carotid pathologies. Furthermore, our logistic regression model demonstrates that those with thalamic lesions had a better functional prognosis than those with lesions in the basal ganglia, which partially agrees with the results of previous studies.

SVO stroke pathophysiology primarily involves lipo-hyalinosis, arteriolosclerosis, and microvascular disease rather than large-vessel atherothrombosis [24]. Carotid plaques are typically associated with macrovascular complications, whereas SVO stroke outcomes might be driven to a greater extent by intracranial small vessel pathology rather than extracranial atherosclerosis [25]. Our results indicate that their direct impact on functional recovery may be limited unless they cause hemodynamic instability or recurrent embolism, even if carotid plaques reflect systemic atherosclerosis. Furthermore, biomarkers of endothelial dysfunction and systemic inflammation should be assessed to determine whether systemic vascular health plays a more significant role in SVO stroke outcomes than localized carotid disease. Furthermore, our finding that diabetes was significantly associated with FD supports the notion that systemic microvascular dysfunction plays a critical role in SVO stroke pathophysiology and recovery. Diabetes is a well-known risk factor for small vessel disease through mechanisms including endothelial dysfunction, chronic inflammation, and accelerated arteriolosclerosis, which can exacerbate cerebral microvascular injury and impair neurovascular repair processes [26,27]. This indirect evidence aligns with the concept that, while carotid plaques represent macrovascular atherosclerosis, the functional outcomes in SVO patients may be more profoundly influenced by systemic microvascular health and metabolic factors such as diabetes [28].

Our rationale for utilizing the American Society of Echocardiography guidelines stemmed from the specific aim of detecting subtle morphological changes relevant to SVO stroke. Unlike the consensus criteria primarily designed to quantify high-grade stenosis in large artery disease [29], the ASE recommendations emphasize CIMT and plaque morphology [14]. We believe that this more holistic approach provides greater insight into the potential mechanisms through which carotid pathologies might influence functional outcomes in SVO, wherein microvascular compromise rather than extensive stenosis often predominates. By incorporating the American Society of Echocardiography criteria, we aimed to account for less severe but clinically meaningful plaque characteristics that might be overlooked, thereby offering a more comprehensive evaluation of carotid disease in the context of SVO stroke. The authors of future studies should focus on larger, prospective assessments to validate these findings and further elucidate the role of carotid plaque morphology in predicting functional outcomes after SVO. Incorporating advanced imaging techniques, such as high-resolution magnetic resonance imaging and carotid plaque vulnerability assessment, might provide further insights into the underlying mechanisms associating carotid atherosclerosis with functional recovery in this specific stroke subtype.

It should be noted that our study has certain limitations. First, the retrospective design means that it was not possible to explain the causalities. Moreover, our cohort was derived from a single cerebrovascular-specialty hospital, potentially limiting the generalizability of our findings to broader populations. Although various covariates were adjusted, the possibility of residual confounding cannot be excluded entirely. Future prospective, longitudinal, and multicenter studies, together with serial carotid imaging with more detailed parameters and various functional assessments, including cognition and mood, are required to confirm our findings. Second, the relatively small sample size may limit the statistical power to detect subtle associations. Third, given the Korean Stroke Registry’s data structure, we utilized the outcome of mRS only 3 months after the index stroke; this short-term observation period did not enable us to discuss the long-term effect of carotid pathologies after SVO stroke. Lastly, while we assessed carotid pathologies using B-mode ultrasound, we did not perform high-resolution magnetic resonance imaging or contrast-enhanced ultrasound, which could provide more detailed information on plaque composition, stability, and embolic potentials. Future studies incorporating high-resolution imaging modalities may yield new insights into the true impact of carotid pathologies on SVO stroke recovery. In addition, although carotid ultrasonography was performed according to the standard protocol established by our institution, given that carotid ultrasound is an operator-dependent modality, inter-operator variability could not be controlled entirely and represents a potential limitation of our study.

## 5. Conclusions

The results of this study reveal that carotid pathologies were not associated with 3-month functional outcomes after SVO stroke in our patient cohort. Notably, age, NIHSS at initial presentation, and diabetes were identified as independent predictors of FD after SVO stroke. One particular clinical implication of our results is the importance of considering traditional risk factors such as age, stroke severity, and comorbid diseases in risk treatment planning for patients with SVO stroke rather than considering carotid pathologies. We have provided concrete suggestions for future studies to clarify the effects of carotid pathologies on short-term functional outcomes after SVO stroke. Further in-depth, prospective, multicenter studies are required to conclusively understand the interplay between carotid atherosclerosis, systemic vascular health, and SVO stroke recovery.

## Figures and Tables

**Table 1:** Baseline characteristics.

Variables	FI Group (n = 276)	FD Group (n = 96)	*p*-Value
Age, years	66.6 ± 11.6	72.6 ± 11.7	<0.001
Male, n (%)	93 (33.7)	51 (53.1)	0.001
Onset to arrival, days	2.8 ± 22.1	1.2 ± 1.4	0.229
NIHSS at initial presentation	1.7 ± 1.7	4.5 ± 3.2	<0.001
Previous mRS, n (%)			0.979
0	274 (99.3)	96 (100.0)	
1	2 (0.7)	0 (0.0)	
Admission route, n (%)			0.022
Outpatient clinic	18 (6.5)	0 (0.0)	
Emergency department	258 (93.5)	96 (100.0)	
Ward type, n (%)			0.014
General ward	59 (21.4)	9 (9.4)	
Stroke care unit	217 (78.6)	87 (90.6)	
Abdominal circumference, cm	84.7 ± 9.3	85.9 ± 10.3	0.277
Body mass index, kg/m^2^	23.9 ± 3.0	24.1 ± 4.1	0.739
Side of the stroke lesion, n (%)			0.750
Right	122 (44.7)	47 (49.0)	
Left	137 (50.2)	45 (46.9)	
Bilateral	14 (5.1)	4 (4.2)	
Lesion location, n (%)			0.003
Basal ganglia	35 (13.0)	17 (17.7)	
Thalamic	40 (14.8)	3 (3.1)	
Cortical area	30 (11.1)	7 (7.3)	
Corona radiata	68 (25.2)	39 (40.6)	
Posterior circulation area	67 (24.8)	17 (17.7)	
Multiple area	30 (11.1)	13 (13.5)	
Comorbidities, n (%)			
Coronary artery diseases	28 (10.1)	11 (11.5)	0.866
Atrial fibrillation	13 (4.7)	5 (5.2)	1.000
Hypertension	159 (57.6)	57 (59.4)	0.856
Diabetes	78 (28.3)	35 (36.5)	0.169
Dyslipidemia	245 (88.8)	85 (88.5)	1.000
Previous antiplatelet therapy	32 (11.6)	21 (21.9)	0.021
Ejection fraction, %	60.1 ± 3.8	59.3 ± 5.1	0.164
Carotid plaque grade ^1^, n (%)			0.016
Normal	29 (12.0)	7 (7.7)	
Grade I	36 (14.9)	9 (9.9)	
Grade II	98 (40.5)	28 (30.8)	
Grade III	79 (32.6)	47 (51.6)	
Mean carotid intimal thickness ^1^, mm	0.7 ± 0.1	0.7 ± 0.1	0.060
NIHSS at discharge	0.5 ± 0.9	3.3 ± 2.6	<0.001

^1^ on the index stroke lesion side. Abbreviations: FD, functional dependency; FI, functional independence; mRS, modified Rankin scale; NIHSS, National Institutes of Health Stroke Scale.

**Table 2:** Results of univariable logistic regression models for functional dependency.

Variables	Odds Ratio	95% CI	*p*-Value
Mean carotid intimal thickness (per 1 SD)	1.23	0.97–1.56	0.084
Carotid plaque grade ^1^			
Normal	Reference		
Grade I	1.04	0.34–3.22	0.950
Grade II	1.18	0.49–3.19	0.721
Grade III	2.46	1.05–6.51	0.049

^1^ Grade I, protuberant plaques with CIMT of <1.5 mm; Grade II, either protuberant or diffuse plaques with CIMT ≥ 1.5 mm but <2.5 mm; and Grade III, CIMT of ≥2.5 mm. Abbreviations: CI, confidence interval; SD, standard deviation.

**Table 3:** Results of multivariable logistic regression models for functional dependency.

Variables	Adjusted OR	95% CI	*p*-Value
Carotid plaque grade ^1^			
Normal	Reference		
Grade I	0.07	0.35–3.42	0.906
Grade II	1.06	0.41–2.98	0.913
Grade III	2.07	0.80–5.91	0.149
Age (years)	1.03	1.00–1.05	0.047
Sex (male)	2.31	1.36–3.97	0.002

^1^ Grade I, protuberant plaques with CIMT of <1.5 mm; Grade II, either protuberant or diffuse plaques with CIMT ≥ 1.5 mm but <2.5 mm; and Grade III, CIMT of ≥2.5 mm. Abbreviation: CI, confidence interval; OR, odds ratio.

**Table 4:** Results of multivariable logistic regression models for functional dependency (backward elimination method ^1^).

Variables	Adjusted OR	95% CI	*p*-Value
Age (years)	1.07	1.03–1.10	<0.001
Sex (male)	0.48	0.24–0.93	0.030
NIHSS at initial presentation	1.84	1.55–2.18	<0.001
Ward type (stroke unit care)	0.56	0.59–4.13	0.376
Body mass index (per 1 kg/m^2^)	1.05	0.96–1.16	0.267
Lesion location			
Basal ganglia	Reference		
Thalamic	0.14	0.03–0.79	0.026
Cortical area	1.12	0.27–4.68	0.878
Corona radiata	1.13	0.44–2.91	0.796
Posterior circulation area	0.51	0.17–1.49	0.220
Multiple area	0.63	0.18–2.16	0.462
Ejection fraction (per 1%)	0.98	0.90–1.07	0.625
Comorbidities			
Coronary artery diseases	1.43	0.52–3.93	0.484
Atrial fibrillation	0.68	0.15–3.04	0.613
Hypertension	0.56	0.28–1.13	0.104
Diabetes	2.84	1.37–5.92	0.005
Dyslipidemia	1.33	0.46–3.86	0.596
Previous antiplatelet therapy	1.59	0.65–3.88	0.305

^1^ Initial covariates: age, sex, NIHSS at initial presentation, onset to arrival, ward type, body mass index, side of the stroke lesion, lesion location, ejection fraction, coronary artery diseases, atrial fibrillation, hypertension, diabetes, dyslipidemia, and previous antiplatelet therapy. Abbreviation: CI, confidence interval; OR, odds ratio.

## Data Availability

The raw data supporting the conclusions of this article will be made available by the authors upon request. The data are not publicly available due to legal or ethical reasons.

## Supplementary Materials

The following supporting information can be downloaded at https://www.mdpi.com/article/10.3390/brainsci15070773/s1. Table S1. Initial laboratory findings. Table S2. Carotid pathologies stratified by sex. Table S3. Carotid pathologies stratified by lesion location. Document S1. Korea Stroke Registry Guideline.

## Abbreviations

The following abbreviations are used in this manuscript:

SVO	small vessel occlusion
CIMT	carotid intima–media thickness
mRS	modified Rankin scale
FI	functional independence
FD	functional dependence
NIHSS	National Institutes of Health Stroke Scale
OR	odds ratio
CI	confidence interval
