# Supplementary material for: The Effects of Carotid Pathologies on Short-Term Functional Outcomes After First-Ever Small Vessel Occlusion Stroke
Source: Brain Sci. 2025 Jul 20;15(7):773. doi: 10.3390/brainsci15070773 (PMC12293778; doi:10.3390/brainsci15070773)
Supplement: Supplementary file 1 [file brainsci-15-00773-s001.zip › Document S1.pdf]

Korea Stroke Registry Guideline

(available at www.strokedb.or.kr)

Personal factors

1. Age: Automatically calculated after entering birthday.

2. Sex: Simply fill in

3. Body mass index automatically calculated after entering height and weight.

- Write down known height and weight, or measure and record if they are not known.

4. Abdominal circumference

- Position: standing with feet apart 25-30 cm apart while exhaling in a comfortable standing position, evenly distribute weight
- Measure at the midpoint between the lower ribs and the upper iliac bones.

Stroke-related factors

1. Initial National Institute of Health Stroke Scale (numeric)

- Based only on the neurological symptoms caused by index stroke.

2. Definition of small vessel occlusion type (TOAST classification)

- Clinical presentation: Presence of a traditional lacunar syndrome without any cortical signs.
- Neuroimaging: Normal or demonstrating a brain stem or subcortical hemispheric lesion (< 2.0 cm).
- Supporting evidence: History of diabetes mellitus (DM) or hypertension (HTN).
- Exclusion: Absence of a potential cardiac embolic source.

*Potential cardiac embolic source.

High-risk sources	Medium-risk sources

Mechanical prosthetic valve	Mitral valve prolapse
Mitral stenosis with atrial fibrillation	Mitral annulus calcification
Atrial fibrillation (other than lone atrial fibrillation)	Mitral stenosis without atrial fibrillation
Left atrial/atrial appendage thrombus	Left atrial turbulence (smoke)
Sick sinus syndrome	Atrial septal aneurysm
Recent myocardial infarction (<4 week)	Patent foramen ovale
Left ventricular thrombus	Atrial flutter
Dilated cardiomyopathy	Lone atrial fibrillation
Akinetic left ventricular segment	Bioprosthetic cardiac valve
Atrial myxoma	Nonbacterial thrombotic endocarditis
Infective endocarditis	Congestive heart failure
	Hypokinetic left ventricular segment
	Myocardial infarction (>4weeks, <6months)

3. Circulatory territory (categorical) and involved side (categorical)

- Records responsible lesion of the index stroke.
- If a lesion has invaded diverse territories, it is indicated in multiple.
- Angiography findings can be applied.
- Categories: Anterior circulation, posterior circulation, and both for circulatory territory. Right, left, and bilateral for the involved side.

4. Onset type (categorical) and time to arrival (numeric)

- Clear onset: When the last known normal time (LNT) and the first known abnormal time (FAT) of neurological symptoms are the same before the onset of stroke (LNT=FAT).
- Unclear onset: Wake-up stroke or daytime unwitnessed stroke
- Time to arrival = LNT to hospital arrival

Laboratory findings (numeric) and blood pressure (numeric)

- Laboratory findings are recorded as the results of the first examination after admission and in the case of in-hospital strokes, as the first examination results after the onset of newly

developed symptoms. (Except serum lipide levels)

- For serum lipid level, the measurement should be conducted after an 8 hour-empty stomach.
- Initial blood pressure: record the first blood pressure measured in a stable state.

Comorbidities (Risk factors)

1. Previous stroke

- Based on patient's or family's statements or medical records.
- Only for complete stoke: It is defined as a sudden onset of localized brain dysfunction lasting more than 24 hours.
- Silent “lacunar” infarction is not a target
- Categories: None, ischemic stroke, hemorrhagic stroke, mixed stroke, unknown

2. Coronary artery disease

- Based on patient's or family's statements or medical records.
- History of myocardial infarction.
- Evidence of asymptomatic myocardial infarction and myocardial ischemia on electrocardiogram or echocardiography.
- History of stable or unstable angina.
- History of coronary angioplasty or surgery.

3. Hypertension: If one or more of the following apply

- Categories: None, history of HTN, and diagnosed at admission.
- Having been diagnosed with hypertension in a hospital and having a history of taking antihypertensive drugs (including cases that stopped taking medication). (History of HTN)
- Having been told that high blood pressure should be treated at the hospital, but the patient has never taken any medications. (History of HTN)
- Blood pressure higher than 140/90 was measured more than twice in self-measurement or health checkup even without history of HTN. (History of HTN)
- In case of high blood pressure of 140/90 mmHg or higher twice or more during hospitalization (in case of acute stroke, blood pressure measured twice after neurological stabilization). (Diagnosed at admission)
- Prescribed antihypertensive medications at the time of discharge. (Diagnosed at admission)

4. Diabetes: If one or more of the following apply

- Having been diagnosed with diabetes in a hospital and having a history of taking oral diabetes medication or insulin treatment (including cases that stopped taking medication or insulin treatment). (History of DM)
- Having been told that high blood sugar should be treated at the hospital, but the patient has never taken any medications. (History of DM)
- Fasting plasma blood glucose of 126 mg/dl or higher for more than 8 hours during hospitalization. (Diagnosed at admission)
- HbA1c 6.5 or higher. (Diagnosed at admission)
- Random glucose level of 200 mg/dl or higher with the following symptoms such as polydipsia, polyuria, and unexplained weight loss during hospitalization. (Diagnosed at admission)
- Glucose level is 200 mg/dl or more 2 hours after the 75g oral glucose tolerance test
- Prescribed oral diabetes medications or insulin treatment at the time of discharge.
- In case of acute stroke, blood pressure measured twice after neurological stabilization

5. Dyslipidemia: If one or more of the following apply

- Having been diagnosed with dyslipidemia in a hospital and having a history of taking lipid lowering agents. (History of DL)
- Having been told that high lipid level should be treated at the hospital, but the patient has never taken any medications. (History of DL)
- LDL C ≥100mg/dl with coronary artery disease or coronary artery like disease (diabetes, peripheral artery disease, abdominal aortic aneurysm, and ischemic stroke due to atherosclerosis). (Diagnosed at admission)
- LDL C ≥130mg/dl with at least two risk factors (smoking, hypertension, HDL C <40 mg/dl, family history of premature CHD [CHD in male first degree relative <55 years old; CHD in female <65 years old], Age [≥45 years old for men or ≥55 years old for women]. Diagnosed at admission)
- LDL C ≥160mg/dl with at least one risk factor (as above). (Diagnosed at admission)
- Determined by the test results performed after fasting for at least 8 hours after hospitalization

6. Atrial fibrillation (categorical)

- Having been diagnosed with atrial fibrillation in a hospital. (History of AF)
- When atrial fibrillation is observed during hospitalization with electrocardiogram, echocardiography, or holter monitoring, etc. (Diagnosed at admission)

7. Previous administration of antiplatelet

- It is defined as a case of regular use within the last month.
- Antiplatelet: All kinds of medication categorized as antiplatelet agents including Aspirin, Clopidogrel, Ticlopidine, etc.
