# Supplementary material for: The Effects of Carotid Pathologies on Short-Term Functional Outcomes After First-Ever Small Vessel Occlusion Stroke
Source: Brain Sci. 2025 Jul 20;15(7):773. doi: 10.3390/brainsci15070773 (PMC12293778; doi:10.3390/brainsci15070773)
Supplement: Supplementary file 1 [file brainsci-15-00773-s001.zip › Supplementary tables.pdf]

Table S1. Initial laboratory findings.

Variables	FI group (n = 316)	FD group (n = 56)	*p*-value
White blood cells, 1000/µL	7.5 ± 2.3	7.7 ± 2.5	0.520
Hemoglobin, g/dL	14.0 ± 1.9	13.5 ± 2.0	0.087
Hematocrit, %	42.4 ± 11.3	40.8 ± 5.3	0.103
Platelet, 1000/µL	241.9 ± 68.4	240.7 ± 69.1	0.906
Blood urea nitrogen, mg/dL	16.2 ± 6.9	16.8 ± 6.0	0.592
Creatinine, mg/dL	0.8 ± 0.2	0.8 ± 0.2	0.967
Total cholesterol, mg/dL	195.7 ± 49.9	201.1 ± 51.8	0.456
Triglyceride, mg/dL	163.7 ± 104.1	152.5 ± 105.0	0.462
High-density lipoprotein, mg/dL	48.8 ± 12.7	48.5 ± 12.4	0.852
Low-density lipoprotein, mg/dL	114.5 ± 44.7	125.6 ± 46.7	0.096
HbA1c, %	6.3 ± 1.4	6.5 ± 1.6	0.172
Fasting blood sugar, mg/dL	124.2 ± 40.4	137.7 ± 55.4	0.092

Abbreviations: FD, functional dependency; FI, functional independence.

Table S2. Carotid pathologies stratified by sex.

Variables	Female (n = 144)	Male (n = 228)	*p*-value
Mean carotid intimal thickness, mm	0.7 ± 0.1	0.7 ± 0.1	0.584
Carotid plaque			0.586
Normal	18 (13.6)	18 (9.0)	
Grade I	18 (13.6)	27 (13.4)	
Grade II	49 (37.1)	77 (38.3)	
Grade III	47 (35.6)	79 (39.3)	

Table S3. Carotid pathologies stratified by lesion location.

Variables	BG (n = 52)	CA (n = 37)	CR (n = 107)	PCA (n = 84)	Thalamus (n = 43)	Multiple (n = 43)	*p*-value
Mean carotid intimal thickness, mm	0.7 ± 0.1	0.7 ± 0.1	0.7 ± 0.1	0.7 ± 0.1	0.7 ± 0.1	0.7 ± 0.1	0.624
Carotid plaque							0.219
Normal	4 (8.5)	7 (22.6)	10 (10.0)	6 (8.1)	4 (10.0)	4 (10.5)	
Grade I	7 (14.9)	5 (16.1)	13 (13.0)	9 (12.2)	7 (17.5)	4 (10.5)	
Grade II	21 (44.7)	9 (29.0)	28 (28.0)	35 (47.3)	19 (47.5)	14 (36.8)	
Grade III	15 (31.9)	10 (32.3)	49 (49.0)	24 (32.4)	10 (25.0)	16 (42.1)	

Abbreviations: BG, basal ganglia; CA, cortical area; CR, corona radiata; PC, posterior circulation area.
